# Supplementary material for: Complex‐centric proteome profiling by SEC‐SWATH‐MS
Source: Mol Syst Biol. 2019 Jan 14;15(1):e8438. doi: 10.15252/msb.20188438 (PMC6346213; doi:10.15252/msb.20188438)
Supplement: Supplementary file 8 — Dataset EV7 [file MSB-15-e8438-s008.zip › feature_plots_string/O75694.pdf]

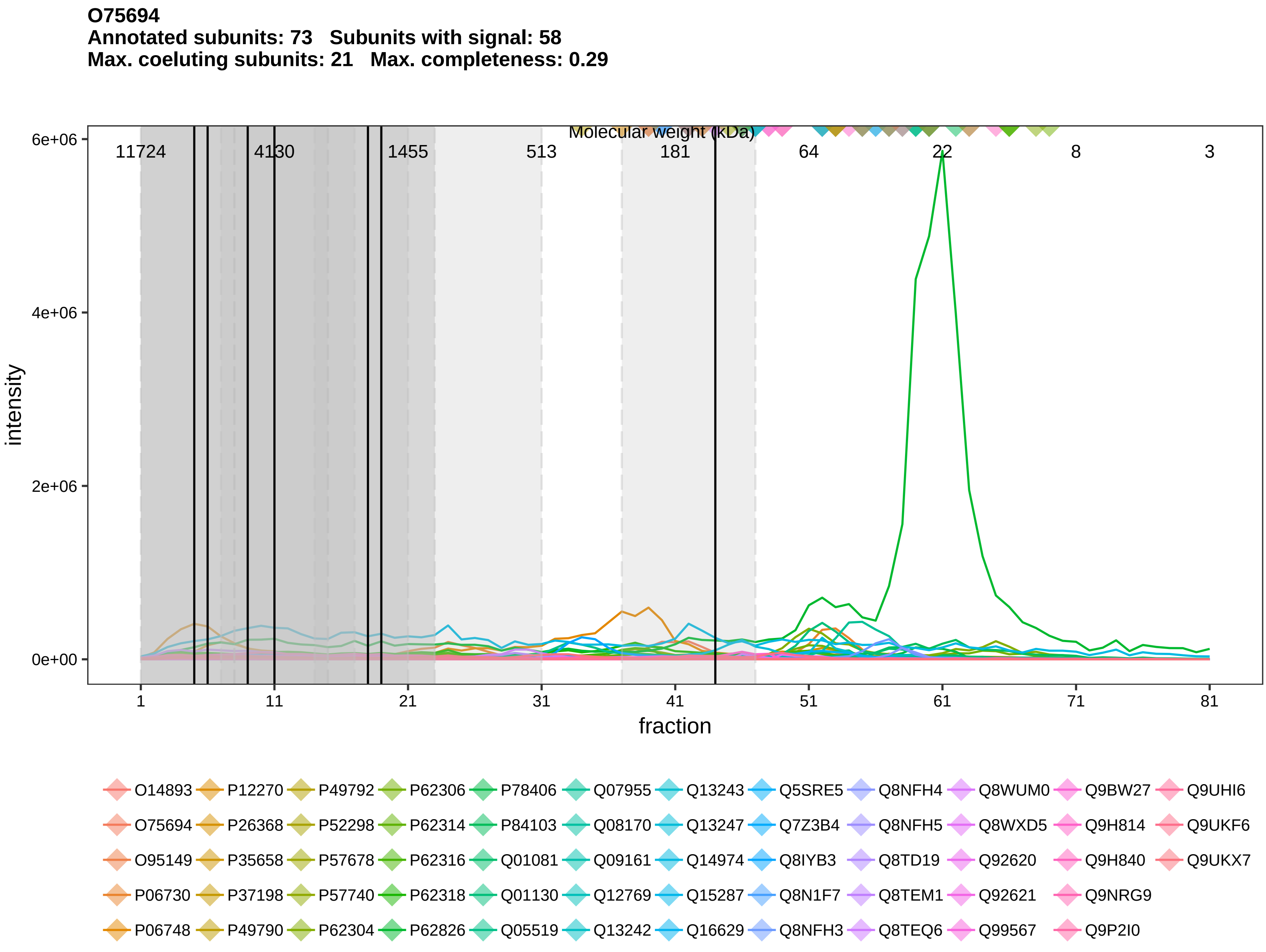
O75694
Annotated subunits: 73 Subunits with signal: 58
Max. coeluting subunits: 21 Max. completeness: 0.29
Molecular weight (kDa)
11724
4130
1455
513
181
64
22
8
3
intensity
6e+06
4e+06
2e+06
0e+00
fraction
1
11
21
31
41
51
61
71
81
O14893
P12270
P49792
P62306
P78406
Q07955
Q13243
Q5SRE5
Q8NFH4
Q8WUM0
Q9BW27
Q9UHI6
O75694
P26368
P52298
P62314
P84103
Q08170
Q13247
Q7Z3B4
Q8NFH5
Q8WXD5
Q9H814
Q9UKF6
O95149
P35658
P57678
P62316
Q01081
Q09161
Q14974
Q8IYB3
Q8TD19
Q92620
Q9H840
Q9UKX7
P06730
P37198
P57740
P62318
Q01130
Q12769
Q15287
Q8N1F7
Q8TEM1
Q92621
Q9NRG9
P06748
P49790
P62304
P62826
Q05519
Q13242
Q16629
Q8NFH3
Q8TEQ6
Q99567
Q9P2I0
